# 33 years of globally calibrated wave height and wind speed data based on altimeter observations

**DOI:** 10.1038/s41597-019-0083-9

**Published:** 2019-05-29

**Authors:** Agustinus Ribal, Ian R. Young

**Affiliations:** 1 Department of Infrastructure Engineering, University of Melbourne, Parkville, Victoria Australia; 2 Department of Mathematics, Faculty of Mathematics and Natural Sciences, Hasanuddin University, Makassar, Indonesia

**Keywords:** Physical oceanography, Environmental impact, Civil engineering

## Abstract

This dataset consists of 33 years (1985 to 2018), of global significant wave height and wind speed obtained from 13 altimeters, namely: *GEOSAT*, *ERS-1*, *TOPEX*, *ERS-2*, *GFO*, *JASON-1*, *ENVISAT*, *JASON-2*, *CRYOSAT-2*, *HY-2A*, *SARAL*, *JASON-3* and *SENTINEL-3A*. The altimeter data have been calibrated and validated against National Oceanographic Data Center (NODC) buoy data. Differences between altimeter and buoy data as a function of time are investigated for long-term stability. A cross validation between altimeters is also carried out in order to check the stability and consistency of the calibrations developed. Quantile-quantile comparisons between altimeter and buoy data as well as between altimeters are undertaken to test consistency of probability distributions and extreme value performance. The data were binned into 1° by 1° bins globally, to provide convenient access for users to download only the regions of interest. All data are quality controlled. This globally calibrated and cross-validated dataset provides a single point of storage for all altimeter missions in a consistent format.

## Abstract (summary)

**Table:** 

Design Type(s)	data integration objective • modeling and simulation objective
Measurement Type(s)	environmental system process
Technology Type(s)	Altimeter Device
Factor Type(s)	temporal_interval • geographic location
Sample Characteristic(s)	Earth (Planet) • ocean

Machine-accessible metadata file describing the reported data (ISA-Tab format)

## Background & Summary

Satellite Radar altimeters have provided global coverage of wind speed and significant wave height (wave height) for more than three decades. Such data have been used for many applications, including: offshore engineering design, validation of numerical models, wind and wave climatology and investigation of long-term trends in oceanographic wind speed and wave height^1–5^. Since the launch of *GEOSAT* in 1985, there has been an almost continuous coverage of global observations from satellite altimeters. Following the conclusion of the *GEOSAT* mission in late-1989, there was a short gap until the launch of the European Remote-Sensing Satellite (*ERS-1*) in mid-1991. Since then, a total of 13 satellite altimeter missions have been operated, with the two latest launches, namely *JASON-3* and *SENTINEL-3A* in 2016. The vast majority of these satellites have been placed in near-polar, sun-synchronous orbits. Altimeters are nadir-looking instruments, meaning they measure along a narrow, pencil-beam directly below the satellite (foot print approx. 10 km wide). This orbit geometry means that the satellites trace-out “herring bone” ground track patterns. Along track resolution of altimeter data is high, with data available at approximately 1 Hz along track (7 km). The ground track separation depends on orbit geometry but can be up to 400 km at the equator, with satellites repeating the same ground tracks on a 3 to 10 day repeat cycle (note that *CRYOSAT-2* has a 369 day repeat cycle with a semi-repeat cycle of 30 days). In recent years, as the number of altimeters in operation has increased, the density of observations has greatly improved.

As a number of agencies have been responsible for the launch and operation of these satellites, data tends to be available from a relatively large number of sites, has been calibrated in a variety of different manners and exists in a variety of data formats. As this obviously complicates usage of the data, a number of attempts have been made to both consistently calibrate altimeter missions but also to provide data repositories for multiple missions. These include: Globwave (http://globwave.ifremer.fr/), Radar Altimeter Data System (RADS, http://rads.tudelft.nl, AVISO (https://www.aviso.altimetry.fr), National Satellite Ocean Application Service (NSOAS, http://www.nsoas.org.cn/) and National Oceanic and Atmospheric Administration (NOAA, https://www.noaa.gov/). However, none of these repositories archives all the missions over the period since 1985 in a consistent manner.

This paper outlines an archive containing wind speed and wave height data, together with related quantities for all 13 altimeter missions. The data are consistently calibrated against buoys, cross-validated between satellites and quality controlled. The satellite calibrations are checked for long term stability, discontinuities and drift. Should any of these occur, they are corrected. Previous satellite data repositories have all stored data along track, following the satellite orbit. Although this format provides a chronological archive of the data, it is generally not optimal for many users. Here, the data are archived in 1° by 1° bins. Within each bin, full data resolution is provided with all parameters for each 1 Hz observation provided (e.g. latitude, longitude, wind speed, wave height, quality flags etc). This binned storage, provides a convenient archiving arrangement for the large datasets, particularly for applications related to specific locations.

This paper describes the calibration, validation, quality control and archiving formats of this comprehensive database. The intention is for the dataset to be dynamic and to grow as future altimeter data become available. It is intended to be updated approximately every six months. This ongoing IMOS (Integrated Marine Observing System) Surface Waves Sub-Facility Altimeter Wave/Wind database is available through the Australian Ocean Data Network portal (AODN: https://portal.aodn.org.au/) the main repository for marine data in Australia. Users can search for the database by entering the search keywords “Altimeter waves” and data can be downloaded using the graphical user interface. The data can be accessed directly from:

http://thredds.aodn.org.au/thredds/catalog/IMOS/SRS/Surface-Waves/Wave-Wind-Altimetry-DM00/catalog.html.

A snapshot of the database at the time of this publication has been assigned a DOI and will be maintained in perpetuity by the AODN^6^. Detailed instructions for accessing the data are provided in the Data Records section below.

## Methods

The calibration and validation methods described below build on the approach adopted by Young *et al*.^7^. The present dataset is, however, much expanded to include more recent altimeter missions. All data have been reprocessed so as to be consistent over the full 33-year data record.

### *In situ* measurements

In order to calibrate the altimeters in a consistent fashion, a long-term high-quality database of buoy *in situ* measurements of wind speed and wave height is required. These data should span a range of different meteorological environments and geographic regions. In addition, such data should be relatively far from land, so as to avoid contamination of altimeter measurements due to land/islands within the altimeter footprint. The only *in situ* dataset which meets these requirements is the National Data Buoy Center (NDBC) buoy archive. Once NDBC data have been quality controlled, the data are archived by the National Oceanographic Data Center (NODC) and are available in the public domain. As with any long term *in situ* data archive of this type there have been changes in buoy hulls, instrumentation packages and analysis methods over the duration of the measurements^8^. The impact of such changes has previously been investigated in the context of trend estimates^9^. In the present application, data from a large number of buoys are pooled and a mean calibration obtained across all buoys. This process ensures that impacts resulting from the changes in hull type at specific locations have a negligible impact on the overall calibration result. The desire to have a long duration dataset relatively far offshore means that the data will be almost exclusively northern hemisphere. Although this will bias mean climatic conditions to some extent, it is unlikely to have a significant impact on the mean calibration^10^. Also, some doubts have been raised about the validity of such data at high wind speeds and wave heights^3,4,8,11–14^. Despite these concerns, the NDBC *in situ* dataset has been extensively used to validate model results and calibrate satellite systems and been found to be of high quality^15,16^.

**Fig. 1:**
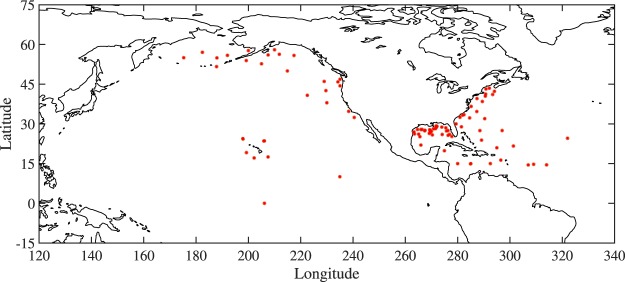
Locations of the NODC buoys (red dots) used in this study in which only buoys more than 50 km offshore are used.

In this work, wind speed and significant wave height have been obtained from NODC moored buoy data more than 50 km offshore to avoid land contamination^17^. From 2011, NODC data contain a series of quality flags for wind speed and significant wave height (0, 1, 2, and 3 which represent *quality_good*, *out_of_range*, *sensor_nonfunctional*, and *questionable*, respectively). Only wind speed and significant wave height which are flagged “0” have been used for calibration of the altimeter data. The locations of NODC buoys used in the calibration are shown in Fig. 1.

The buoys measure either significant wave height (*H*_*s*_), wind speed at the height of the anemometer *z* (*U*_*z*_) or both. For wind speed, a consistent reference height of 10 m is required (*U*_10_). This was obtained by assuming a neutral stability logarithmic boundary layer as given by^1^:

1 $${U}_{10}={U}_{z}\sqrt{\frac{{\kappa }^{2}}{{C}_{d}}}\,\frac{1}{ln\left(z/{z}_{0}\right)},$$

where *κ* is the von Kármán constant which is approximately 0.4, *C*_*d*_ is the drag coefficient and *z*_*o*_ is the roughness length. Measurements of *C*_*d*_ over the ocean yield results with scatter over an order of magnitude, and much research has focused on the wind speed and sea state dependence of *C*_*d*_^1,18,19^. In this work, *C*_*d*_ = 1.2 × 10^−3^ and *z*_*o*_ = 9.7 × 10^−5^ m are used. As mentioned in previous studies^7^, a different assumption of *C*_*d*_ does not have a significant influence on the final satellite wind speed^17^. For a more detailed description of NOAA buoy data, one can refer to Zieger^20^. This choice of boundary layer correction is consistent with previous altimeter calibrations^17^.

### Altimeter data

**Fig. 2:**
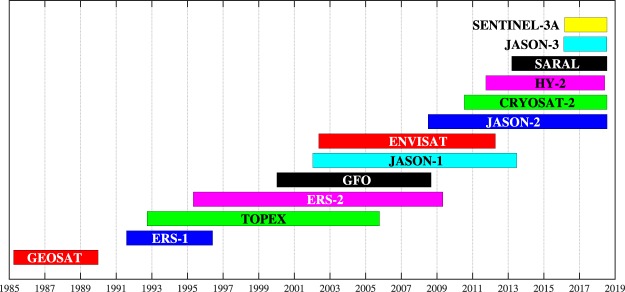
Durations of altimeter data in the database from all satellite missions.

The altimeter data used in this database were sourced from three different archived locations, namely Globwave^21^, Radar Altimeter Data System (RADS)^22^, and National Satellite Ocean Application Service (NSOAS)^23^. A total of 13 satellite missions: *GEOSAT*, *ERS-1*, *TOPEX*, *ERS-2*, *GEOSAT Follow-On (GFO)*, *JASON-1*, *ENVISAT*, *JASON-2*, *CRYOSAT-2*, *Hai Yang-2A (HY-2A)*, *Satellite with ARgos* and *ALtika (SARAL)*, *JASON-3* and *SENTINEL-3A* (expressed in the order of launch) were included. The duration of the combined altimeter missions is 33 years, from 1985 until 2018 except for the short break from 1990 until the middle of 1991 as shown in Fig. 2.

The data from six altimeters which were mostly retired before 2013, namely *GEOSAT*, *ERS-1*, *TOPEX*, *ERS-2*, *GFO*, and *ENVISAT* were obtained from Globwave. The calibration and validation of these altimeters has previously been described, using essentially the same process adopted here^7^. These altimeters were re-calibrated for the present database, resulting in very minor changes to the calibration relationships.

Data for a further six altimeters: *JASON-1*, *JASON-2*, *CRYOSAT-2*, *SARAL*, *JASON-3* and *SENTINEL-3A* were obtained from RADS. The final satellite is *Hai Yang*-*2A* (*HaiYang* means ocean in Chinese). This satellite is China’s first dynamic environmental satellite and the data from this altimeter is not available in the public domain. However, following personal communication, the data were provided from NSOAS. Summary information for each of the satellites/altimeters is included in Table 1.

**Table 1:** Summary of altimeter operating characteristics for the thirteen altimeter missions, including exact repeat mission period (time until satellite repeats the same ground track), orbit parameters, antenna properties, latitude coverage, and operational time for which data is available.

Altimeter	Exact repeat mission (days)	Inclination	Altitude (km)	Freq. (GHz)	Freq. Band	Latitude coverage	Initial Date	Final Date
*GEOSAT*	23/17	108°	800	13.5	Ku	−73 to 72	31/03/1985	31/12/1989
*ERS-1*	35/168	98°	784	13.8	Ku	−81.5 to 81.5	01/08/1991	02/06/1996
*TOPEX*	10	66°	1336	13.575 5.3	Ku C	−66 to 66	25/09/1992	08/10/2005
*ERS-2*	35	98°	784	13.8	Ku	−81.5 to 81.5	29/04/1995	11/05/2009
*GFO*	17	108°	800	13.5	Ku	−73 to 72	07/06/2000	07/09/2008
*JASON-1*	10	66°	1336	13.575 5.3	Ku C	−66.15 to 66.15	15/01/2002	21/06/2013
*ENVISAT*	35	98°	784	13.6 3.2	Ku S	−82 to 82	14/05/2002	08/04/2012
*JASON-2*	10	66°	1336	13.575 5.3	Ku C	−66.15 to 66.15	04/07/2008	Ongoing
*CRYOSAT-2*	30	92°	717	13.575	Ku	−88 to 88	14/07/2010	Ongoing
*HY-2A*	14	99.3°	963.6	13.58 5.25	Ku C	−81 to 80	01/10/2011	06/06/2018
*SARAL*	35	98.538°	~800	35.75	Ka	−81.49 to 81.49	14/03/2013	Ongoing
*JASON-3*	10	66°	1336	13.575 5.3	Ku C	−66.15 to 66.15	12/02/2016	Ongoing
*SENTINEL-3A*	27	98.65°	814.5	13.575 5.41	Ku C	−78 to 81	01/03/2016	Ongoing

Values of significant wave height and wind speed are determined from the high frequency altimeter data by fitting a functional form to the radar return from the ocean surface in a process called waveform retracking. As noted above, the original data for the present database were sourced from Globwave, RADS and NSOAS. In the case of both Globwave^24^ and RADS^25^, these data were originally sourced from the various satellite agencies in the form of 1 Hz Geophysical Data Records (GDRs). The processing used to form the GDRs uses a range of different retracking approaches and no attempt has been made to harmonize the retracking. Rather, we use the 1 Hz data from the GDRs and calibrate at this level. This calibration clearly removes some differences between various datasets. A harmonized retracking of all data would presumably further increase the quality of data but is beyond the scope of this database.

### Altimeter quality controls

Altimeter, Geophysical Data Records are not free from data errors and such data contain numerous data “spikes” due to land and ice contamination and issues associated with variable quality of the altimeter waveform received by the satellite^17^. As indicated previously^7^, the Globwave data contains a series of quality flags (0, 1, and 2, representing *good_measurement*, *acceptable_for_some_applications*, and *bad_measurement*, respectively)^24^. These flags proved very reliable in excluding poor quality data.

In the present database, a series of data flags defined as 1, 2, 3, 4, and 9 represent *Good_data*, *Probably_good_data*, *SAR-mode data or possible hardware error* (only used for *CRYOSAT-2*), *Bad_data* and *Missing_data*, respectively, have been used. Hence, the quality flags from Globwave have been transformed from flags 0, 1 and 2 to the present flags 1, 2 and 4. Moreover, all *NaN* values in Globwave have been defined as missing data which are flagged 9.

The RADS and NSOAS data do not have a similar system of quality flags. Hence, the following criteria have been used to assess the quality of the data. This approach is similar to that adopted previously^17,26^. Initially the *H*_*s*_ data are considered:

1. If *H*_*s*_ > 30 m, then the data point was flagged “4” which means “bad” data.
2. All points identified as over land and ice using land/ice masks (as defined in GDRs distributed with the original data by the various satellite agencies) were discarded.
3. In the present dataset, 1 Hz values are used. However, the satellite agencies do distribute data related to variability of 20 Hz products in the GDRs. All data in which the standard deviation of these 20 Hz altimeter data values is greater than 2.5 m signifies data where there is significant variability within the footprint and were flagged “4”.
4. After applying these quality controls, the data were divided into blocks of 25 points, which represents approximately 180 km along the ground track. As argued in Zieger *et al*.^17^, this represents segments long enough to form reliable statistics but not so long that data will not be coherent. Individual values in the block were identified as outliers, and flagged “4”, based on the median absolute deviation (MAD)^27^. The MAD is defined as^28^:
  $$MAD=b\,median\left\{\left|\,{x}_{i}-{M}_{n}\,\right|\right\},$$
  where ${M}_{n}=median\left\{{x}_{i}\right\}$ and *x*_*i*_ is the original observation in which *i* = 1, 2, 3, … *n*. In this case n = 25. The value of *b* is given by 1.4826 which is the scaling factor of Gaussian distributions^29^. Furthermore, following Miller^30^, a threshold value of 3 has been chosen and hence all values which are outside the following criterion were categorized as outliers^27^. The criterion is given by
  $${M}_{n}-3\times MAD < {x}_{i} < {M}_{n}+3\times MAD.$$
  This equation can be rewritten as:
  $$\left|\frac{{x}_{i}-{M}_{n}}{MAD}\right| < 3.$$
5. In a final check, blocks identified in test 4 above, were further considered. These blocks were re-divided into sub-blocks, either side of flagged points. These sub-blocks were considered in the following manner:

- Each sub-block was examined for outliers, as in test 4, with any further points failing the test flagged as erroneous (“4”).
- If the ratio $R={\sigma }_{{H}_{s}}\left(block\right)/{\bar{H}}_{s}\left(block\right)$ is large, where ${\sigma }_{{H}_{s}}(block)$ is the standard deviation of the block and ${\bar{H}}_{s}(block)$ is the mean of the block, then it indicates that it is possible that there are multiple spikes in the block. If *R* > 0.5, then the entire sub-block was flagged as “4”.

The steps undertaken at points 4 and 5 above are intended to flag erroneous “spikes” in the data. Visual examination of many cases indicated it was remarkably successful at this, whilst not removing strong along-track gradients which may be caused by strong currents^31,32^. Nevertheless, the data are retained and flagged as “4”. Users interested in along-track variability can process such data if desired.

Similar criteria have been applied for wind speed (*U*_10_). In this case, for test 1 the wind speed limit was set at 60 ms^−1^ for all altimeters except *SARAL*. In the case of *SARAL* this limit was set at 24 ms^−1^. (See discussion of *SARAL* calibration below.) As for significant wave height, wind speed values above these limits were classified as “bad” data which are flagged “4”.

### Calibration against *in situ* measurements

The quality controlled significant wave height and wind speed data were calibrated by comparing the buoy measurements with altimeter passes. Buoy observations and altimeter passes were considered a “matchup” if they satisfied the following criteria:

- Altimeter track was within 50 km of the buoy and the overpass occurred within 30 min. of the buoy recording data. These matchup criteria have been widely used in previous studies^17,33–38^.
- Only buoys which are more than 50 km offshore have been considered in order to avoid the impact of the proximity of land on both buoy and satellite observations.
- A minimum of five points were required in the altimeter pass within the 50 km radius region around the buoy.
- Any large variability in the along-track altimeter data was excluded. Specifically, passes in which $\sigma \left({H}_{s}\right)/{\bar{H}}_{s} > 0.2$ were excluded, where *σ*(*H*_*s*_) and ${\bar{H}}_{s}$ are the standard deviation and mean of values of the altimeter data within the 50 km radius region around the buoy.

Again, the same criteria were also applied for wind speed. As not all buoys measure both wind speed and wave height, there is not a one to one overlap between buoys used to calibrate wind speed and wave height.

The values of significant wave height (*H*_*s*_) for calibration can be extracted directly from the various data archives. However, altimeter *U*_10_ is calculated from the radar cross-section, *σ*_0_ (ratio of the returned to transmitted energy of the altimeter pulse) and a variety of different relationships have historically been used for different altimeters. In order to have consistent calibrations across the various altimeters, it is desirable to use a consistent *U*_10_ − *σ*_0_ relationship^7,17^. Hence, following the method used in Zieger *et al*.^17^ and Young *et al*.^7^, uncalibrated wind speed was calculated based on the backscatter coefficient *σ*_0_ using the algorithm^39^:

2 $${U}_{10}={U}_{m}+1.4{U}_{m}^{0.096}exp(-0.32{U}_{m}^{1.096})$$

where

3 $${U}_{m}=\left\{\begin{array}{cc}\alpha -\beta {\sigma }_{0} & for\,{\sigma }_{0}\le {\sigma }_{b}\\ \gamma exp\left(-\delta {\sigma }_{0}\right) & for\,{\sigma }_{0} > {\sigma }_{b}\end{array}\right.$$

The values of $\alpha ,\,\beta ,\,\gamma ,\,\delta ,\,and\,{\sigma }_{b}$ in equation (3) are given by

4 $$\alpha =46.5,\,\beta =3.6,\,\gamma =1690,\,\delta =0.5,\,{\sigma }_{b}=10.917\,dB.$$

Note that the units of wind speed in the above relationships are meters per second and the radar backscatter decibels.

Equations (2) and (3) with the value of the parameters given in (4) have been developed for Ku-band radar altimeters (13.5–13.8 GHz). The *SARAL* altimeter, however, is a Ka-band radar altimeter (35.75 GHz). Following Lillibridge *et al*.^40^ for calibrated Ka altimeters, (2) and (3) still hold but with the coefficients in (4) given by:

5 $$\alpha =34.2,\,\beta =2.48,\,\gamma =720,\,\delta =0.42,\,{\sigma }_{b}=11.4\,dB.$$

In addition, for high wind speed where *U*_10_ > 18 ms^−1^, a modified relationship^41^ has been adopted which is given by

6 $${U}_{10}=-6.4{\sigma }_{0}+69,\,if\,Eq.\,(2) > 18\,{ms}^{-1}.$$

This high wind speed relationship has recently been validated for use in extreme value analyses^42^. Quilfen *et al*.^43^ also obtained a very similar result to Eq. (6). There is some evidence that altimeter wind speed may be a function of sea state in addition to radar cross-section^44^. The analysis here does not consider any sea state dependence, which appears small.

**Fig. 3:**
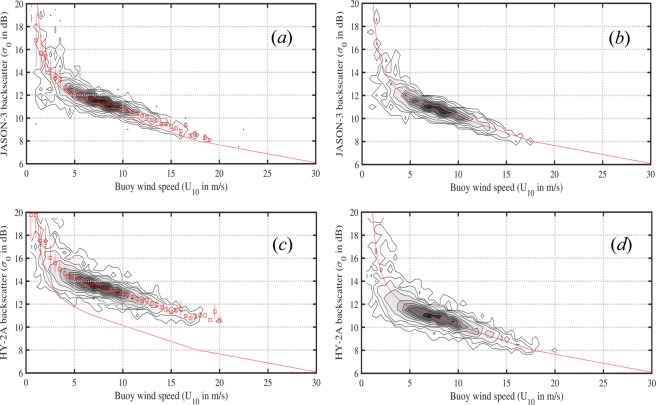
Comparisons between buoy wind speed and radar cross section for *JASON-3* and *HY-2A*. (**a**) Recorded radar cross section for *JASON-3*. (**b**) *JASON-3* comparison after ***σ***_0_ was adjusted by −0.569 dB. (**c**) Recorded radar cross-section for *HY-2A*. (**d**) *HY-2A* comparison after ***σ***_0_ was adjusted by −2.605 dB.

Values of radar cross-section (*σ*_0_) provided for each of the altimeter systems have a variety of different datum offsets. Therefore, following Young *et al*.^7^, it is necessary to remove this offset before applying (3)–(6). This is achieved by comparing buoy measurements of *U*_10_ with altimeter *σ*_0_ and determining the offset *σ*_*offset*_ which gives the best fit (in a least-squares sense) between the data and (3)–(6). Figure 3 shows this process for *JASON-3* and *HY-2A* (the altimeter with the largest *σ*_0_ offset, see Online-only Table 1).

A linear regression analysis is then performed between the buoy and altimeter match-up data (*U*_10_ values). Although the buoy data are considered “ground truth” for the purposes of the calibration, such data does contain both sampling and calibration errors^7^. As a consequence, a conventional regression analysis is not appropriate. However, in such cases, a reduced major axis (RMA) regression can be used^45^. This regression minimizes the triangular area bounded by the vertical and horizontal offsets between the data point and the regression line and the cord of the regression line. This is in contrast to a conventional regression which minimizes the vertical axis offset from the regression line. In addition, standard least squares regression analysis is highly sensitive to outliers. Such outliers can be removed by the use of robust regression^46^. Robust regression assigns a weight to each point, with values between 0 and 1. Points with a value less than 0.1 were designated as outliers and removed from the analysis before applying the RMA regression analysis.

In the type of regression analysis described above, it is desirable to have as many matchups as possible, as this reduces the confidence limits on the calibration (regression) result. Hence, it is usual to pool data from all buoys over the full duration of the altimeter mission^7,17^. However, such an “average” calibration will mask any changes in the calibration over time (drift or discontinuities in calibration). Such issues can be addressed by firstly calibrating the individual altimeters against all buoy data (average calibration) and then examining the differences between buoy and altimeter (with average calibration) as a function of time^7^.

**Fig. 4:**
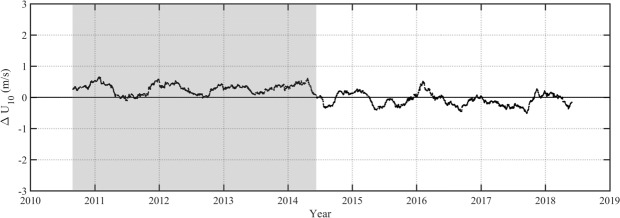
Difference between *CRYOSAT-2* and NODC buoy values of *U*_10_ as a function of time when a single calibration relation is used over the full period of the mission.

Young *et al*.^7^, identified a number of such changes to calibration in their analysis of *GEOSAT*, *ERS-1*, *TOPEX*, *ERS-2*, *GFO* and *ENVISAT*. For the seven additional altimeters included here, discontinuities in the significant wave height of *HY-2A* and the wind speed of *CRYOSAT-2* (see Fig. 4) were identified. Other altimeters do not change their calibration significantly during their respective missions. Note that the present results for *HY-2A* are consistent with the results of Liu *et al*.^47^.

**Fig. 5:**
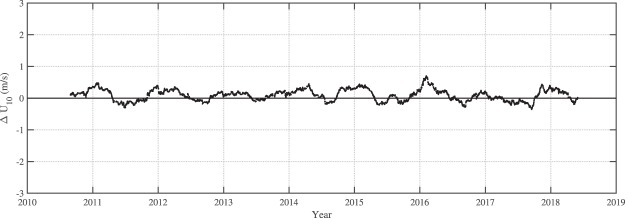
Difference between *CRYOSAT-2* and NODC buoy values of *U*_10_ as a function of time when a piecewise calibration was used.

Figure 4 shows the difference between buoy and altimeter values of wind speed, Δ*U*_10_ as a function of time for *CRYOSAT-2*. As can be seen in this figure, a clear change in calibration occurs in mid-2014. When such discontinuities were identified in the data, a piecewise calibration was performed. That is, the altimeter was calibrated separately either side of the discontinuity. Figure 5 shows the result, once the data were calibrated in this fashion, clearly removing the discontinuity.

In Figs 4 and 5 there is a clear periodicity in the data with an annual signal in Δ*U*_10_. As demonstrated in Young and Donelan^10^ this is a result of changes in the structure of the atmospheric boundary layer as a result of changes in the air-water temperature difference (atmospheric stability). These stability effects do not impact *H*_*s*_ and no attempt has been made to correct *U*_10_ for this effect.

**Fig. 6:**
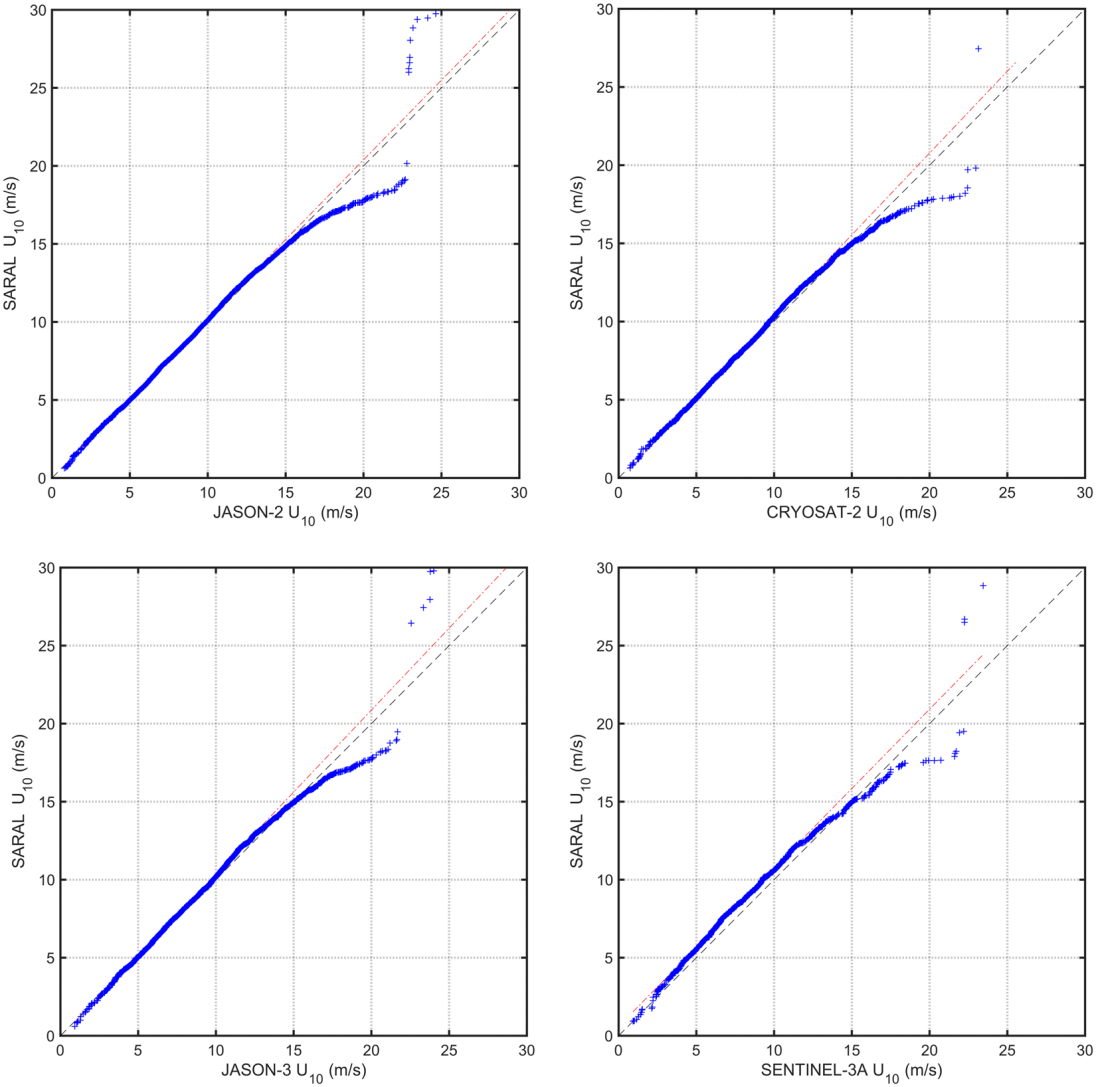
Q – Q plots of cross-validation between *SARAL* and *JASON-2*, *JASON-3*, *CRYOSAT-2*, and *SENTINEL-3A*.

As noted earlier, *SARAL* operates in the Ka frequency band, whereas all other altimeters operate in the Ku-band. As a consequence, the parameters in the *U*_10_ − *σ*_0_ relations (2) and (3) were defined by (5). Examination of scatter and Q − Q plots between buoy and *SARAL* wind speeds showed good agreement using this approach. However, when cross-validation was carried out with other altimeters (see Technical validation below) and the wind speed range was extended to higher values, it was clear that the *SARAL* calibration under-estimated higher wind speeds. This behavior is shown in the altimeter-altimeter validation Q − Q plots in Fig. 6.

In order to address this issue, the other calibrated altimeters in orbit at the same time as *SARAL* (*JASON-2*, *JASON-3*, *CRYOSAT-2*, and *SENTINEL-3A)* were used to determine a wind speed correction for *SARAL* wind speed *U*_10_ > 10 ms^−1^. The wind speed correction developed is quadratic (see Online-only Table 1). The available data for the correction was limited to *U*_10_ < 24 *ms*^−1^. As caution should be exercised in a quadratic extrapolation, wind speeds which are greater than 24 ms^−1^ have been flagged as “bad”. As a result, the calibration relation for *SARAL* wind speed has been separated into two regions – *U*_10_ < 10 ms^−1^ and 10 *ms*^−1^ ≤ *U*_10_ ≤ 24 *ms*^−1^. The final calibration relationships for significant wave height are shown in Online-only Table 2 and for wind speed in Online-only Table 1.

## Data Records

A total of 26 parameters, as outlined in Table 2 are archived in the repository^6^. Again, since SARAL is a Ka-band altimeter, rather than Ku-band, the variable names have been changed accordingly. All data are stored in NetCDF files with the records binned into 1° by 1° bins. Within each bin, full data resolution is provided with the recorded latitude, longitude of every 1 Hz measurement recorded. The binned storage provides a convenient mechanism for most users to access data. Data files are not included for areas where there is no data - land or ice areas or bins where the satellite orbit meant there were no overpasses. Individual files are provided for each of the 13 altimeters as summarized in Table 3. Data commences in 1985 and continues to 2018 except for the short break in 1990 and 1991. It should be noted that although data in the nearshore region is provided in the database, it is recommended for applications more than 50 km offshore. Data less than 50 km offshore which passed all other quality control processes are flagged “2” – “probably good data”.

**Table 2:** List of all parameters in the database.

No.	NetCDF variable name	Description
1	TIME	Time
2	LATITUDE	Latitude
3	LONGITUDE	Longitude
4	BOT_DEPTH	Bathymetry
5	DIST2COAST	Distance to coast
6	SIG0_C	C-band altimetry backscatter coefficient
7	SIG0_C_quality_control	C-band altimetry backscatter coefficient flags
8	SIG0_C_num_obs	Number of valid 20 Hz C-band altimetry backscatter coefficient measurements making up the 1 Hz measurement
9	SIG0_C_std_dev	Standard deviation of the 20 Hz C-band altimetry backscatter coefficient data making up the 1 Hz measurement
10	SIG0_KU	Ku-band altimetry backscatter coefficient
11	SIG0_KU_quality_control	Ku-band altimetry backscatter coefficient flags
12	SIG0_KU_num_obs	Number of valid 20 Hz Ku-band altimetry backscatter coefficient measurements making up the 1 Hz measurement
13	SIG0_KU_std_dev	Standard deviation of the 20 Hz Ku-band altimetry backscatter coefficient data making up the 1 Hz measurement
14	SWH_C	Uncalibrated C-band altimetry significant wave height
15	SWH_C_quality_control	Quality flag for C-band altimetry significant wave height
16	SWH_C_num_obs	Number of valid 20 Hz C-band altimetry measurements of significant wave height making up the 1 Hz measurement
17	SWH_C_std_dev	Standard deviation of the 20 Hz C-band altimetry significant wave height data making up the 1 Hz measurement
18	SWH_KU	Uncalibrated Ku-band altimetry significant wave height
19	SWH_KU_CAL	Calibrated Ku-band altimetry significant wave height
20	SWH_KU_quality_control	Quality flag for Ku-band altimetry significant wave height
21	SWH_KU_num_obs	Number of valid 20 Hz Ku-band altimetry measurements of significant wave height making up the 1 Hz measurement
22	SWH_KU_std_dev	Standard deviation of the 20 Hz Ku-band altimetry significant wave height data making up the 1 Hz measurement
23	UWND	ECMWF model zonal wind speed
24	VWND	ECMWF model meridional wind speed
25	WSPD	Uncalibrated wind speed based on wind function
26	WSPD_CAL	Calibrated wind speed based on wind function

**Table 3:** The number of files for each altimeter and total file size.

No.	Altimeter name	Number of files	File size (Gigabytes)
1	*CRYOSAT-2*	45,483	8.39
2	*ENVISAT*	42,600	8.37
3	*ERS-1*	42,765	6.22
4	*ERS-2*	42,585	7.71
5	*GEOSAT*	37,756	4.63
6	*GFO*	36,866	5.95
7	*HY-2A*	42,089	7.07
8	*JASON-1*	35,810	9.85
9	*JASON-2*	34,783	8.66
10	*JASON-3*	28,395	4.23
11	*SARAL*	41,582	6.46
12	*SENTINEL-3A*	41,990	5.64
13	*TOPEX*	36,042	8.44
**Total**		**508,746**	**91.62**

All data files are provided in NetCDF format following the IMOS (Integrated Marine Observing System) data protocols upon which the project is based^48,49^. The IMOS standard flag system is used for all data flags – where 1, 2, 3, 4, and 9 represent *Good_data*, *Probably_good_data*, *SAR-mode altimeter data or hardware error* (*CRYOSAT-2* only), *Bad_data* and *Missing_data*, respectively. Note that *CRYOSAT-2* operated for some geographic regions in SAR-mode. This data has been flagged as “3” in the database. The calibrations developed for *CRYOSAT-2* were not developed for SAR-mode data and hence, this data should be used with caution. The filenames follow the format:

$${\bf{IMOS}}\mbox{--}{\bf{SRS}} \mbox{-} {\bf{Surface}} \mbox{-} {\bf{Waves}}\mbox{--}{\bf{MW}}\mbox{--}{\bf{ALTIMETER}}\mbox{--}{\bf{FV02}}\mbox{--}{\bf{Lat}}\mbox{--}{\bf{Lon}}\mbox{--}{\bf{DM00}}\,.{\bf{nc}}$$

where

- **IMOS**: name of the project.
- **SRS-Surface-Waves**: representing the present facility.
- **MW**: M signifies meteorological related parameters and W signifies wave related parameters.
- **ALTIMETER**: name of altimeter (variable).
- **FV02**: representing the version of the file.
- **Lat**: latitude north or south of the most southern border of the 1° file (variable).
- **Lon**: the longitude of the most western border of the 1° file (variable).
- **DM00**: first version of delayed mode product.

As the full database consists of approximately 500,000 files (Table 3), it has been stored using the following hierarchy for folders:

\Satellite_Name\20degree_by_20degree_subregion\NetCDF_files (as above)

e.g. \JASON1\020S_280E\IMOS_SRS-Surface-Waves_MW_JASON-1_FV02_016S-282E-DM00.nc.

For both the 20-degree subregion and the 1 deg NetCDF file the latitude, longitude signifies the west most and south most locations for the data region.

The data can be accessed in the number of ways:

- Static archive
  A static “snapshot” of the data as described in this paper has been archived and allocated the identifier 10.26198/5c77588b32cc1. This is a full copy of all data at the date of submission of this publication.
- Dynamic archive – AODN graphical portal

As the intention is to update the data at approximately 6-month intervals, a dynamic archive is maintained at the Australian Ocean Data Network (AODN). The AODN portal can be accessed at: https://portal.aodn.org.au/. The user can access the data graphically from the portal. To find the data, the following navigation is recommended.

(i) Click the *“Get Ocean Data Now”* button
(ii) Scroll to the keyword search box at the bottom left of the screen. Enter the keywords *“altimeter waves”*.
(iii) Click on the thumbnail map of the world to the right. The graphical interface which opens allows the user to scroll to any area of the world and define a region to download with the mouse. The specific satellites to download can be specified in the menu to the left.

(c) Dynamic archive – Direct interface

The dynamic archive can also be accessed directly as an Amazon S3 archive. It is recommended that this is done using software such as Cyberduck. Instructions to set up such a server can be found at:

https://help.aodn.org.au/downloading-data-from-servers/amazon-s3-servers/

Once access to the S3 server is gained, the user should navigate to:

*IMOS/SRS/Surface-Waves/*

The dynamic archive is in the folder: *Wave-Wind-Altimetry-DM00*

The static archive mentioned above is in the folder:

*Wave-Wind-Altimetry-DM00_C-20190228T030000Z*

## Technical Validation

As noted above, a further set of checks to verify the consistency and stability of various altimeters was conducted in the form of cross validations between altimeter missions. The same criteria as for the buoy matchups have been applied for the cross validations (observations within 50 km and 30 min). Again, RMA regression has been performed for each cross validation. Matchup scatter plots, probability density functions as well as Q – Q plots were analyzed for each combination. This follows the same approach used by Young *et al*.^7^.

**Fig. 7:**
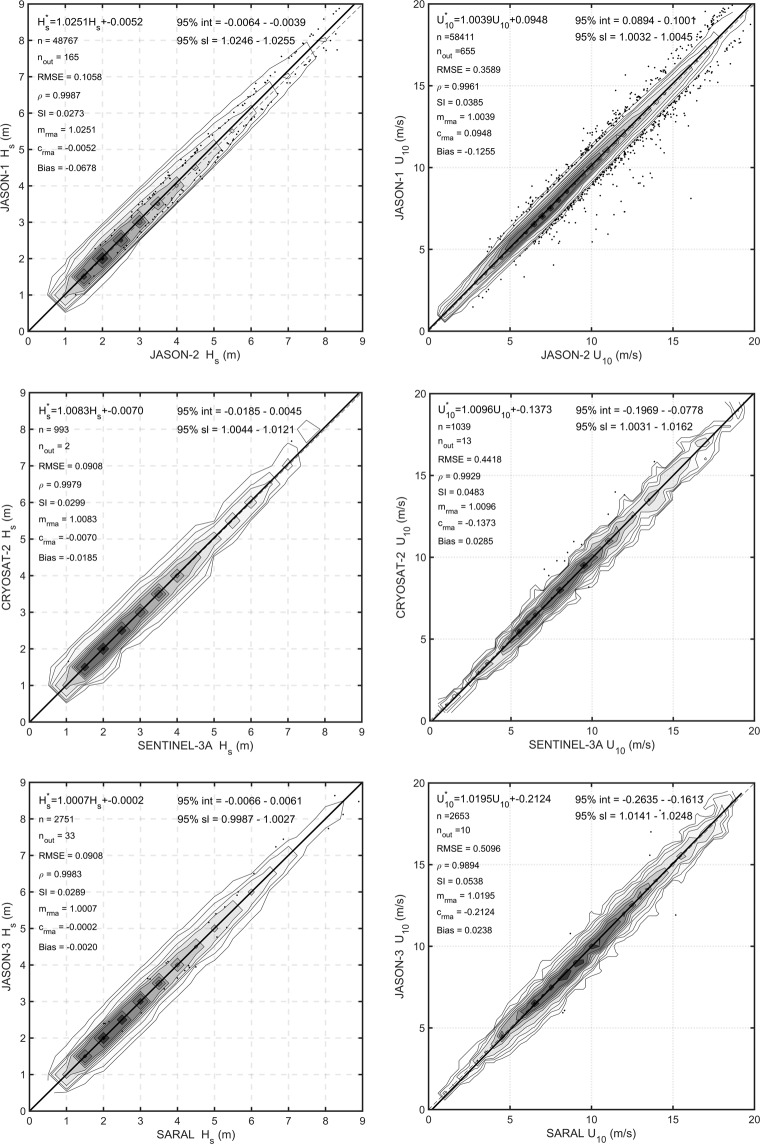
Cross-validation matchup plots between the altimeters for significant wave height and wind speed. Shown are the 1:1 agreement (dashed diagonal line) and the RMA regression (thick solid line). Contours show the density of matchup data points which has been normalized such that the maximum value is 1.0. Contours are drawn at 0.9, 0.8, 0.7, …, 0.1, 0.05, 0.025, 0.01.

**Fig. 8:**
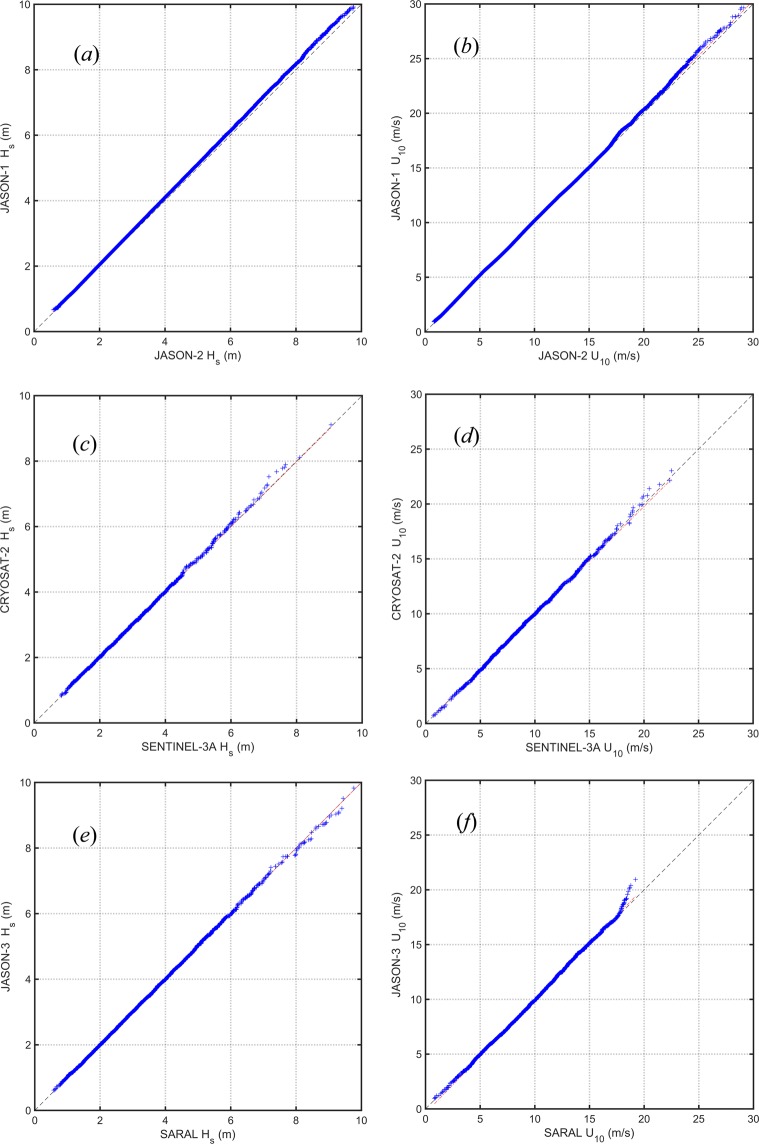
Q − Q plots between the altimeters for significant wave height and wind speed. Panels (**a,c,e**) refer to wave height and (**b**,**d**,**f**) to wind speed. (**a**,**b**) *JASON-2* – *JASON-1*, (**c**,**d**) *SENTINEL-3A* – *CRYOSAT-2* and (**e**,**f**) *SARAL* – *JASON-3*.

**Fig. 9:**
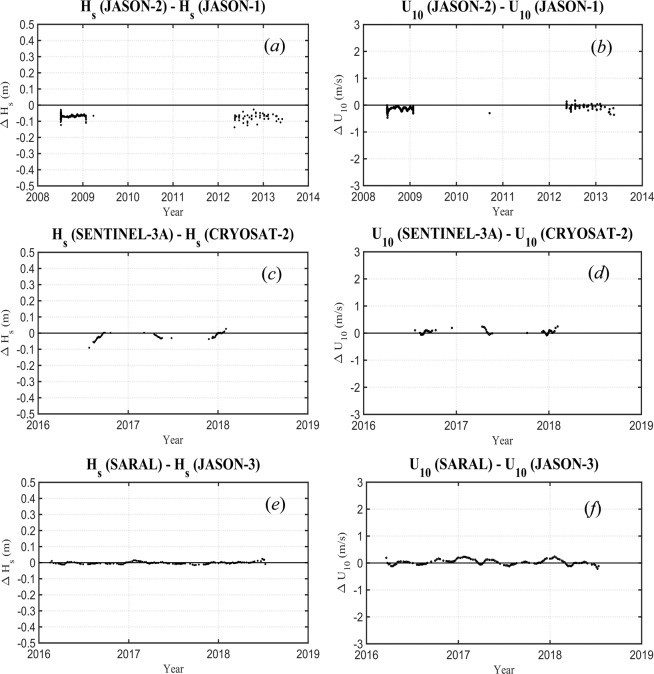
Altimeter – Altimeter difference for significant wave height and wind speed as a function of time. Panels (**a,c,e**) refer to wave height and (**b**,**d**,**f**) to wind speed. (**a**,**b**) *JASON-2* – *JASON-1*, (**c**,**d**) *SENTINEL-3A* – *CRYOSAT-2* and (**e**,**f**) *SARAL* – *JASON-3*.

There are a large number of combinations of satellite matchups across the 13 altimeter missions. Three cases are shown below as examples (*JASON-1 − JASON-2*, *CRYOSAT-2 − SENTINEL-3A*, *JASON-3 − SARAL*). In each case, calibrated data are shown. Figure 7 shows RMA cross-validation, Fig. 8 presents Q − Q plots and Fig. 9 shows altimeter - altimeter differences as a function of time. Note that the gaps in the time series in Fig. 9 occur due to changes in orbit of satellites over time. Such changes mean that for a period of time there will be no cross-over points which meet the match-up criteria required. Data are shown for both *H*_*s*_ and *U*_10_. As calibrated data are used, the scatter plots (Fig. 7) and Q − Q plots (Fig. 8) should lie along the diagonal and the altimeter – altimeter differences should be zero.

In order to analyse the performance of the cross validations, four different statistical parameters, namely bias (*B*), root-mean square error (*RMSE*), Pierson’s correlation coefficient (*ρ*) and scatter index (*SI*) were used. These parameters were calculated based on the following relations^16^ in which *M* and *O* stand for model and observation, respectively.

7 $$B=\frac{1}{N}\sum _{i=1}^{N}\left({M}_{i}-{O}_{i}\right)$$

8 $$RMSE=\sqrt{\frac{1}{N}\sum _{i=1}^{N}{({M}_{i}-{O}_{i})}^{2}}$$

9 $$SI=\frac{\sqrt{\frac{1}{N}\sum _{i=1}^{N}{\left({M}_{i}-{O}_{i}-B\right)}^{2}}}{\frac{1}{N}\sum _{i=1}^{N}{O}_{i}}$$

10 $$\rho =\frac{cov(M,O)}{\sqrt{cov(M)cov(O)}}$$

A summary of the statistical parameters is provided in Tables 4 to 7. It should be noted that *C-2*, *HY2*, *J-1*, *J-2*, *J-3*, *SA*, and *S-3* are abbreviated forms of *CRYOSAT-2*, *Hai Yang-2A*, *JASON-1*, *JASON-2*, *JASON-3*, *SARAL* and *SENTINEL-3A*, respectively. Moreover, whilst other statistical parameters are commutative, bias is not. Hence, in order to read the bias in Tables 6 and 7, one has to read it from row to column.

**Table 4:** *RMSE* and correlation coefficient for significant wave height cross-validation.

Alt	C-2	HY2	J-1	J-2	J-3	SA	S-3	
C-2		0.993	0.997	0.997	0.997	0.997	0.998	***ρ***
HY2	0.157		0.995	0.992	0.993	0.989	NaN	
J-1	0.195	0.179		0.999				
J-2	0.119	0.181	0.106		0.999	0.998	0.998	
J-3	0.118	0.181		0.076		0.998	0.997	
SA	0.112	0.097		0.092	0.091		0.996	
S-3	0.091	NaN		0.131	0.132	0.112		
***RMSE***

**Table 5:** *RMSE* and correlation coefficient for wind speed cross-validation.

Alt	C-2	HY2	J-1	J-2	J-3	SA	S-3	
C-2		0.922	0.993	0.992	0.994	0.986	0.993	***ρ***
HY2	1.558		0.901	0.949	0.968	0.923	NaN	
J-1	0.489	1.959		0.996				
J-2	0.489	1.551	0.359		0.999	0.990	0.995	
J-3	0.443	1.258		0.182		0.989	0.995	
SA	0.601	0.929		0.500	0.510		0.991	
S-3	0.442	NaN		0.462	0.406	0.683		
***RMSE***

**Table 6:** Scatter index and bias for significant wave height cross-validation.

Alt	C-2	HY2	J-1	J-2	J-3	SA	S-3	
C-2		−0.012	−0.118	−0.033	−0.008	0.005	0.019	***B***
HY2	0.049		−0.107	−0.018	0.005	−0.011	NaN	
J-1	0.048	0.045		0.068				
J-2	0.037	0.053	0.027		0.023	0.027	−0.048	
J-3	0.037	0.053		0.024		0.002	0.028	
SA	0.037	0.045		0.028	0.029		0.013	
S-3	0.030	NaN		0.039	0.042	0.051		
**Scatter Index (SI)**

**Table 7:** Scatter index and bias for wind speed cross-validation.

Alt	C-2	HY2	J-1	J-2	J-3	SA	S-3	
C-2		0.485	−0.125	−0.127	−0.126	−0.105	−0.029	***B***
HY2	0.161		−0.992	−0.960	−0.762	−0.161	NaN	
J-1	0.050	0.180		0.126				
J-2	0.050	0.123	0.039		0.083	0.023	0.212	
J-3	0.045	0.110		0.018		−0.024	0.096	
SA	0.065	0.139		0.052	0.054		0.463	
S-3	0.048	NaN		0.042	0.042	0.057		
**Scatter Index (SI)**								

All the statistical parameters shown in Tables 4 to 7 indicate consistent performance between the altimeters. All values of wave height correlation coefficient (Table 4) are close to one. Similarly, *RMSE* values (Table 4) are small, with all values less than 20 cm. Bias and scatter index (Table 6) are also very small, being less than 12 cm and 0.1, respectively. Similar to the cross-validation for the significant wave height, the cross-validation for wind speed indicates excellent agreement, with the exception of *HY-2A* (Tables 5 and 7). Although the *HY-2A* wind speed data are clearly of lower quality than the other altimeters, it is still likely that it will be acceptable for most applications. *SENTINEL-3A* is a new-generation SAR mode altimeter and operates in this mode at all times. As a result, the radar return is no longer Gaussian, which can introduce biases due to swell and relative track angle to swell direction^50^. The results in Tables 4 to 7 indicate that this platform produces error statistics comparable to the other platforms averaged over all geographic regions. It is possible, however, that its performance in swell-dominated regions may differ from the other platforms. These potential regional differences have not been explored in the present work. It should be noted that *NaN* values in the tables indicate that the corresponding cross-validations do not satisfied our matchups criteria. It does not mean, they do not have any matchups.

A number of other studies have used this dataset for global studies. Young and Donelan^10^ have examined global climatology of *U*_10_ and *H*_*s*_ and found the data to be consistent with buoy and model reanalysis results. Takbash *et al*.^42^ have used the data to examine global extreme value wind speed and wave height (i.e. 1 in 100 year values). They show that the data produces extreme value estimates consistent with buoy data. That study indicates that although the present calibrations are limited to values of *U*_10_ < 24 ms^−1^ and *H*_*s*_ < 9 m the tails of the respective probability distribution functions remain valid above these limits. Young and Ribal^51^ have used the data to investigate trends in wind speed and wave height. This is a particularly demanding analysis, as it requires long term stability of the data. The results show that wind speed and wave height trends are consistent in both magnitude and spatial distribution and that the wind speed trends are consistent with radiometer and scatterometer data.

### ISA-Tab metadata file

Download metadata file

## Data Availability

All data are available as NetCDF files. The calibration process described in this paper and the production of the NetCDF files were undertaken using Matlab scripts written for this purpose. This code is available from the corresponding author upon request.

## Online-only Tables

**Online-only Table 1:** Calibration relationships for wind speed, obtained from the RMA regression. ${{\boldsymbol{U}}}_{10}^{* }$ is the calibrated value and ***U***_10_ the uncalibrated data. Also shown are the confidence limits on the regression, number of points, *n* and the percentage of outliers from the robust regression. *σ*_*offset*_ is the offset to be applied to the *U*_10_ − *σ*_0_ relationship

Altimeter	Period	Calibration Relation	95% limit Slope	95% limit Offset	*n*	%Out
*GEOSAT*	31 Mar 85 – 30 Dec 89	${U}_{10}^{* }=1.064{U}_{10}-0.897$	1.001 to 1.127	−1.373 to −0.422	575	0.7
		$${\sigma }_{offset}=0.152dB$$				
*ERS-1*	1 Aug 91 – 2 Jun 96	${U}_{10}^{* }=1.017{U}_{10}-0.222$	0.0990 to 1.044	−0.430 to −0.014	2625	1.6
		$${\sigma }_{offset}=0.104dB$$				
*TOPEX*	25 Sep 92 – 8 Oct 05	${U}_{10}^{* }=1.009{U}_{10}-0.177$	0.995 to 1.024	−0.290 to −0.064	8618	1.6
		$${\sigma }_{offset}=-0.601dB$$				
*ERS-2*	29 Apr 95 – 1 Jan 00	${U}_{10}^{* }=1.002{U}_{10}+0.064$	0.975 to 1.029	−0.136 to 0.264	2918	1.4
	1 Jan 00 – 1 Jan 01	${U}_{10}^{* }=0.974{U}_{10}-0.214$	0.922 to 1.026	−0.634 to 0.206	650	1.5
	1 Jan 01 – 1 Apr 01	${U}_{10}^{* }=0.889{U}_{10}-0.297$	0.748 to 1.030	−1.813 to 1.223	126	0.8
	1 Apr 01 – 1 Jun 05	${U}_{10}^{* }=0.963{U}_{10}-0.103$	0.938 to 0.988	−0.299 to 0.093	2878	1.6
	1 Jun 05 – 11 May 09	${U}_{10}^{* }=1.008{U}_{10}-0.053$	0.983 to 1.034	−0.235 to 0.129	3075	1.2
		$${\sigma }_{offset}=0.040dB$$				
*GFO*	7 Jan 00 – 1 Jan 01	${U}_{10}^{* }=0.963{U}_{10}-0.286$	0.861 to 1.065	−0.963 to 0.391	200	2.0
	1 Jan 01 – 1 Mar 06	${U}_{10}^{* }=1.068{U}_{10}-0.185$	1.040 to 1.095	−0.395 to 0.025	2900	2.5
	1 Mar 06 – 7 Sep 08	${U}_{10}^{* }={U}_{10}-0.0025t+0.2$	—	—	—	—
		$${\sigma }_{offset}=-0.361dB$$				
*JASON-1*	15 Jan 02 – 21 Jun 13	${U}_{10}^{* }=0.969{U}_{10}+0.121$	0.961 to 0.978	0.056 to 0.185	6530	0.7
		$${\sigma }_{offset}=-0.681dB$$				
*ENVISAT*	14 May 02 – 8 Apr 12	${U}_{10}^{* }=1.025{U}_{10}-0.245$	1.011 to 1.039	−0.354 to −0.137	9972	2.0
		$${\sigma }_{offset}=-0.056dB$$				
*JASON-2*	04 Jul 08 – present	${U}_{10}^{* }=0.993{U}_{10}-0.004$	0.985 to 1.001	−0.057 to 0.064	7423	1.0
		$${\sigma }_{offset}=-0.329dB$$				
*CRYOSAT-2*	14 Jul 10 – 10 Jun 14	${U}_{10}^{* }=1.050{U}_{10}-0.314$	1.033 to 1.067	−0.440 to −0.187	1954	1.7
		$${\sigma }_{offset}=0.040dB$$				
	10 Jun 14 – present	${U}_{10}^{* }=1.016{U}_{10}-0.144$	1.003 to 1.030	−0.246 to −0.042	2648	1.2
		$${\sigma }_{offset}=-0.104dB$$				
*HY-2A*	01 Oct 11 – 06 Jun 18	${U}_{10}^{* }=1.024{U}_{10}-0.241$	1.008 to 1.040	−0.357 to −0.126	3394	1.3
		$${\sigma }_{offset}=-2.605dB$$				
*SARAL*	14 Mar 13 – present	For ${U}_{10} < 10\,{ms}^{-1}$	1.043 to 1.072	−0.479 to −0.268	2719	1.2
		$${U}_{10}^{* }=1.057{U}_{10}-0.374$$				
		$${\sigma }_{offset}=-0.233dB$$				
		For ${U}_{10}\ge 10\,{ms}^{-1}$				
		$${U}_{10}^{* }=a{U}_{10}^{2}+b{U}_{10}+c$$				
		$$a=0.084,\;b=1.006$$				
		$$c=11.840$$				
*JASON-3*	12 Feb 16 - present	${U}_{10}^{* }=0.998{U}_{10}-0.030$	0.982 to 1.013	−0.148 to 0.088	2031	1.6
		$${\sigma }_{offset}=-0.569dB$$				
*SENTINEL -3A*	01 Mar 16 - present	${U}_{10}^{* }=1.013{U}_{10}-0.106$	0.996 to 1.029	−0.229 to 0.018	1796	2.0
		$${\sigma }_{offset}=-0.104dB$$				

**Online-only Table 2:** Calibration relationships for significant wave height, obtained from the RMA regression. ${{\boldsymbol{H}}}_{{\boldsymbol{s}}}^{* }$ is the calibrated value and ***H***_*s*_ the uncalibrated data. Also shown are the confidence limits on the regression, number of points, *n* and the percentage of outliers from the robust regression

Altimeter	Period	Calibration Relation	95% limit Slope	95% limit Offset	*n*	%Out
*GEOSAT*	31 Mar 85 – 30 Dec 89	${H}_{s}^{* }=0.962{H}_{s}+0.058$	0.924 to 0.997	−0.035 to 0.141	467	3.5
*ERS-1*	1 Aug 91 – 2 Jun 96	${H}_{s}^{* }=1.167{H}_{s}+0.118$	1.139 to 1.195	0.053 to 0.183	1578	2.4
*TOPEX*	25 Sep 92 – 25 Apr 97	${H}_{s}^{* }=1.055{H}_{s}-0.063$	1.039 to 1.072	−0.093 to −0.033	2402	1.5
	25 Apr 97 – 30 Jan 99	$$\begin{array}{l}{H}_{s}^{* }={H}_{s}+0.0303-\\ 0.0542{[exp(0.0027t)]}^{1.1080}\end{array}$$	—	—	—	—
	30 Jan 99 – 8 Oct 05	${H}_{s}^{* }=1.056{H}_{s}-0.063$	1.047 to 1.066	−0.093 to −0.033	5110	1.7
*ERS-2*	29 Apr 95 – 11 May 09	${H}_{s}^{* }=1.081{H}_{s}+0.026$	1.067 to 1.094	−0.064 to 0.116	5658	2.0
*GFO*	7 Jan 00 – 7 Sep 08	${H}_{s}^{* }=1.077{H}_{s}+0.087$	1.066 to 1.088	−0.002 to 0.176	4533	1.8
*JASON-1*	15 Jan 02 – 21 Jun 13	${H}_{s}^{* }=1.057{H}_{s}-0.060$	1.052 to 1.061	−0.069 to −0.051	6736	1.6
*ENVISAT*	14 May 02 – 1 Aug 04	${H}_{s}^{* }=1.013{H}_{s}+0.099$	0.977 to 1.037	0.051 to 0.148	1078	2.3
	1 Aug 04 – 8 Apr 12	${H}_{s}^{* }=0.995{H}_{s}+0.098$	0.986 to 1.005	0.047 to 0.150	6506	2.1
*JASON-2*	04 Jul 08 – present	${H}_{s}^{* }=1.032{H}_{s}-0.069$	1.027 to 1.036	−0.078 to 0.060	6946	1.8
*CRYOSAT-2*	14 Jul 10 – present	${H}_{s}^{* }=0.977{H}_{s}-0.081$	0.972 to 0.983	−0.093 to −0.069	4110	1.9
*HY-2A*	01 Oct 11 – 01 Apr 13	${H}_{s}^{* }=1.085{H}_{s}+0.092$	1.068 to 1.101	0.058 to 0.125	759	2.1
	15 Apr 13 – 06 Jun 18	${H}_{s}^{* }=1.075{H}_{s}-0.040$	1.066 to 1.083	−0.057 to −0.023	2529	1.8
*SARAL*	14 Mar 13 – present	${H}_{s}^{* }=1.009{H}_{s}-0.086$	1.004 to 1.014	−0.096 to −0.076	2906	2.2
*JASON-3*	12 Feb 16 – present	${H}_{s}^{* }=1.029{H}_{s}-0.079$	1.021 to 1.036	−0.094 to −0.065	1901	1.7
*SENTINEL-3A*	01 Mar 16 – present	${H}_{s}^{* }=0.948{H}_{s}+0.103$	0.940 to 0.957	0.086 to 0.121	1409	1.8
