# Publisher Correction: 33 years of globally calibrated wave height and wind speed data based on altimeter observations

**DOI:** 10.1038/s41597-019-0108-4

**Published:** 2019-06-19

**Authors:** Agustinus Ribal, Ian R. Young

**Affiliations:** 1 Department of Infrastructure Engineering, University of Melbourne, Parkville, Victoria, Australia; 2 Department of Mathematics, Faculty of Mathematics and Natural Sciences, Hasanuddin University, Makassar, Indonesia

**Keywords:** Physical oceanography, Environmental impact, Civil engineering

Correction to: *Scientific Data* 10.1038/s41597-019-0083-9, published online 29 May 2019

A number of errors in the content and formatting of Online-only Table 1 and Online-only Table 2 in the original version of this Data Descriptor were introduced during the typesetting process. These errors have now been corrected.

